# Enhancing germination and growth of canola (*Brassica napus* L.) through hydropriming and NaCl priming

**DOI:** 10.1038/s41598-024-63948-2

**Published:** 2024-06-18

**Authors:** Rahila BiBi, Nosheen Noor Elahi, Subhan Danish, Tahani Awad Alahmadi, Mohammad Javed Ansari

**Affiliations:** 1 Institute of Botany, Bahauddin Zakariya University, Multan, Punjab Pakistan; 2 Pesticide Quality Control Laboratory, Old Shujabad Road, Multan, Punjab Pakistan; 3 Department of Pediatrics, College of Medicine and King, Khalid University Hospital, King Saud University, Medical City, PO Box-2925, 11461 Riyadh, Saudi Arabia; 4 Department of Botany, Hindu College Moradabad (Mahatma Jyotiba Phule Rohilkhand University Bareilly), Moradabad, India

**Keywords:** Chlorophyll content, Growth attributes, Canola plants, Seed priming, Oleic acid, Plant sciences, Plant stress responses, Abiotic

## Abstract

The excessive accumulation of sodium chloride (NaCl) in soil can result in soil salinity, which poses a significant challenge to plant growth and crop production due to impaired water and nutrient uptake. On the other hand, hydropriming (WP) and low level of NaCl priming can improve the germination of seeds, chlorophyll contents, oil and seed yield in plants. That’s why this study investigates the impact of hydro and different levels of NaCl (0.5, 1.0, 1.5 and 2.0%) priming, as pre-treatment techniques on canola seeds germination, growth and yield of two varieties Punjab and Faisal Canola. Results showed that, WP performed significant best for increase in germination (~ 20 and ~ 22%) and shoot length (~ 6 and ~ 10%) over non-priming (NP) in Punjab Canola and Faisal Canola respectively. A significant increase in plant height (~ 6 and ~ 7%), root length (~ 1 and ~ 7%), shoot fresh weight (~ 5 and ~ 7%), root fresh weight (~ 6 and ~ 7%) in Punjab Canola and Faisal Canola respectively. It was also observed that plants under WP and 0.5%NaCl priming were also better in production of seed yield per plant, oil contents, silique per plant, seeds per silique, and branches per plant chlorophyll contents and leaf relative water contents over NP. In conclusion, WP and 0.5%NaCl has potential to improve the germination, growth, yield and oil attributes of canola compared to non-priming, 1.0%NaCl priming, 1.5%NaCl priming and 2.0%NaCl priming.

## Introduction

Abiotic stress includes heavy metals, drought, water logging, cold, heat, and salinity. It reduces the production and cultivation of crops^1–5^. Salinity is the foremost detrimental factor that severely limits and causes a substantial reduction in crop quality and productivity^6^. Salinity is the salt accumulation on soil and near the soil surface. Globally, it affected 836 Mha of cultivated land^7^. It is reported that more than 50% of the world’s cultivated land will be affected by salinity by 2050^8^. Salinity impacts plant functions, including morphology, biochemistry, metabolism, and ultrastructural. It hinders nutrient uptake, leading to poor root systems, ionic toxicity, and osmotic stress^9^. It reduces plant growth, germination capacity, and sugar content. Elevated salinity also affects metabolic functioning^10^, inhibits chlorophyll biosynthesis, and disrupts reactive oxygen species balance^11^. Crops in semi-arid and arid environments are at higher salinity risk, necessitating improved methods to enhance growth and productivity.

Canola (*Brassica napus* L.) is generally an edible rape seed^12^. It is an essential industrial crop belonging to the Brassicaceae family and is the third largest oil crop growing worldwide after soya bean and palm^13^. Canada is the largest producer of *Brassica napus* L. and globally generate a yield of 50% of vegetable oil for domestic purpose^14^. It is utilized as a source of biofuel, human food, or animal forage. Cultivating canola benefits soil fertility by incorporating canola in crop rotation for good agricultural practices. Compared to 2004, canola production increased by 61% in 2019^15^.

Seed priming is the controlled hydration of seeds immersed in a low osmotic potential solution. It is a popular, inexpensive, and easy-to-use technique that enhances the germination percentage and reduces physiological heterogeneity in seed masses, mainly under a stressful environment^16,17^. Several seed priming techniques are reported in the literature, like hydro priming, halo priming, and osmo-priming to improve seed germination under unfavorable environments^18^. During seed priming, physiological situations may stimulate growth and germination. According to^19^, seed priming increases the growth and yield of soybeans. Previously, extensive literature was available on the direct effect of salinity on crop plants, but limited literature was available on the response of plants upon seed priming with NaCl.

Hence, the current study has examined the effect of different levels of NaCl and water seed priming on the growth and germination of canola. The study is covering the knowledge gap regarding use of low levels of NaCl as priming agent and its comparison with established hydropriming technique. The aims of study were improvement of germination, growth and yield attributes of canola plants via use of priming technique. The novelty aspect of current study lies in the use of NaCl as priming agent for improvement of canola growth, yield and oil contents. It is hypothesized that low level of NaCl and hydropriming may pose beneficial effect to the growth and germination of plants.

## Material and methods

### Experimental design and treatment plan

There were six treatments: Control (non-primed), hydroprimed (WP), 0.5% NaCl primed, 1% NaCl primed, 1.5% NaCl primed and 2% NaCl primed. All the treatments were applied in 6 replicates. For laboratory and pot studies the design of experiment was completely randomized design (CRD).

### Priming conditions

Canola seeds were primed for 24 h under 20 °C along with continuous aeration using different concentrations of NaCl (0.5%, 1%. 1.5%, 2%) and deionized water (hydropriming). Non-primed seeds were used as a control.

### Seed collection

Canola (*Brassica napus* L.) varieties (Faisal and Punjab canola) were collected from the Ayyub Agricultural Research Center in Faisalabad, Pakistan. The experiment was conducted at the Botanical Garden of Bahauddin Zakariya University.

### Pot dimensions and details

A plastic pot with a diameter of 28 cm and 12 kg of soil, prepared by mixing 1 proportion of debris and 2:2 proportion of sand and soil, was used to sow the remaining seeds of each treatment. Pots were continuously watered to maintain the moisture of 65% field capacity.

### Data collection

The germination rate was calculated based on seedling germination. After 40 days of germination, plants were harvested, and leaf relative water content, carotenoids, chlorophyll a, b, and total chlorophyll content were determined. Following a 50-day growth period post-sowing, the plants were harvested to gather data. Immediately after harvest, measurements were taken for plant height, shoot and root lengths and fresh weights. To determine the shoot and root dry weights, samples were subjected to oven drying at 65 °C for 72 h until a constant weight was achieved.

### Chlorophyll contents, and carotenoids,

To quantify the chlorophyll a, chlorophyll b, and total chlorophyll levels in freshly harvested wheat leaves, we followed a procedure adapted from Arnon’s method^20^. The extraction process involved the use of an 80% acetone solution. Absorbance measurements were recorded at distinct wavelengths: 663 nm for chlorophyll a, 645 nm for chlorophyll b, 470 nm for carotenoids, and 530 nm for anthocyanins.

### Leaf Relative water content

Following the protocol of^21^, leaf relative water content was measured. Briefly, fresh leaves were taken uniformly sized, and fresh weight (FW) was determined. Leaves were submerged in distilled water for 24 h and were weighed again (TW). Afterward, leaves were kept in place for drying for 48 h, and dry weight was taken (DW). Then, leaf relative content was calculated using the given formula.

$${\text{RWC}}\,\left( {\text{\%}} \right) = \frac{{{\text{FW}} - {\text{DW}}}}{{{\text{TW}} - {\text{FW}}}} \times 100$$

### Determination of yield and oil attributes

To determine the yield, different morphological parameters, including seed yield per plant, seeds per silique, silique length, branches per plant, and seed output per plant, were measured soon after harvesting the plants.

### Oil contents

Seeds were allocated to the Nuclear Institute for Food and Agriculture for oil testing (NIFA) to determine the oil percentage, linoleic acid, moisture content, and oleic acid. Using NIR technology.

### Statistical analysis

The data were subjected to standard statistical analysis to facilitate comparison. Treatment significance was assessed using a Two-way ANOVA, and the comparison between treatments was carried out through paired comparisons utilizing the Tukey test at a significance level of *p* ≤ 0.05. To visualize the data, generate cluster plots with convex hulls, hierarchical cluster plots, and calculate Pearson correlations, the Origin software^22^ was used.

### Ethical approval

We all declare that manuscript reporting studies do not involve any human participants, human data, or human tissue. So, it is not applicable. Study protocol must comply with relevant institutional, national, and international guidelines and legislation. Our experiment follows the with relevant institutional, national, and international guidelines and legislation.

### Permissions or licenses

Permissions or licenses were obtained or required to collect the seed.

## Results

### Germination, plant height, shoot, and root length

**Figure 1:**
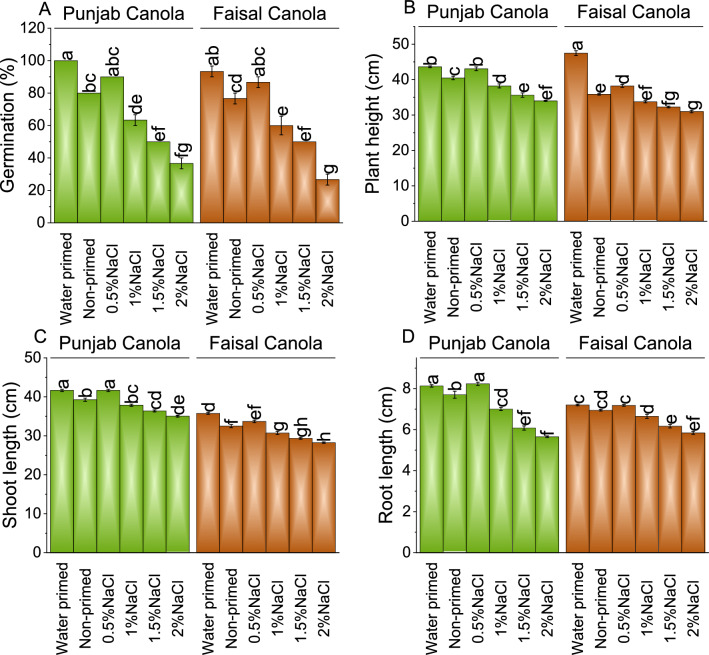
Effect of different pre-treatments of NaCl on germination (**A**), plant height (**B**), shoot length (**C**), and root length (**D**) of Punjab Canola and Faisal Canola. Bars are means of 6 replicates ± SE. Difference letters on bars showed significant changes at *p* ≤ 0.05; Tukey test.

Punjab Canola seeds with WP showed 100%, while 80% germination with NP, representing ~ 20% increase in germination percentage WP compared to NP. At 0.5% NaCl, Punjab Canola seeds showed a ~ 10% decrease over WP seeds and a ~ 13% increase compared to NP. Under 1.0, 1.5, and 2.0% NaCl priming, germination decreased by ~ 37, ~ 50, and ~ 63% from WP, and ~ 21, ~ 38, and ~ 54% than NP, respectively. In Faisal Canola, WP ~ 22% higher germination percentage compared to NP seeds. Under 0.5% NaCl stress, Faisal Canola seeds displayed ~ 7% decrease from WP and ~ 13% increase in germination over NP. In the case of 1.0, 1.5, and 2.0% NaCl, a decline of ~ 36, ~ 46, and ~ 71% compared to WP, while ~ 2, ~ 35, and ~ 65% than NP, respectively was observed in germination percentage (Fig. 1A).

At 0.5% NaCl, the plant height showed ~ 1% decrease compared to WP plants, but a ~ 6% increase compared to NP in Punjab Canola. However, at 1.0, 1.5, and 2.0% NaCl seed priming, the plant height declined up to ~ 12, ~ 18, and ~ 22% compared to WP, and ~ 6, ~ 12, and ~ 16% compared to NP, respectively in Punjab Canola. In contrast, Faisal Canola plant height was ~ 32% higher in WP than NP. At 0.5% NaCl, WP Faisal Canola showed decrease of ~ 19% compared to WP, but a ~ 7% increase over NP. Under 1.0, 1.5, and 2.0% NaCl, the plant heights was declined ~ 29, ~ 32, and ~ 35% than WP, and ~ 6, ~ 10, and ~ 14% compared to NP, respectively (Fig. 1B).

Punjab Canola seeds treated with WP exhibited a ~ 6% increase in shoot length compared to NP. Under 0.5% NaCl priming, Punjab Canola plants maintained similar shoot lengths to WP but displayed a ~ 6% increase over NP. However, shoot length was decreased ~ 9, ~ 13, and ~ 16% in 1.0, 1.5 and 2.0% NaCl priming compared to WP in Punjab Canola. Similarly, a decline of ~ 4, ~ 7 and ~ 11% in shoot length was noted over NP where 1.0, 1.5 and 2.0% NaCl priming was done in Punjab Canola. For Faisal Canola WP plants displayed a ~ **10%** increase in shoot length compared to NP. Compared to WP in Faisal Canola, ~ 6, ~ 14, ~ 18 and ~ 21% decrease in shoot length was noted in 0.5, 1.0, 1.5 and 2.0% NaCl seed priming. It was noted that under 1.0, 1.5 and 2.0% NaCl seed priming caused ~ 5, ~ 10 and ~ 13% decrease while 0.5% NaCl seed priming resulted in ~ 4% increase in shoot length in Faisal Canola (Fig. 1C).

Results showed that 0.5% NaCl seed priming caused ~ 1 and ~ 7% increase in root length over WP and NP, respectively, in Punjab Canola. In Faisal Canola, 0.5% NaCl seed priming showed ~ 0.3 and ~ 3% enhancement in root length than WP and NP, respectively. However, treatments 1.0, 1.5 and 2.0% NaCl seed priming caused a decrease in root length ~ 15, ~ 25 and ~ 30% in Punjab Canola while ~ 8, ~ 14 and ~ 19 in Faisal Canola compared to WP. Similarly, 1.0, 1.5 and 2.0% NaCl seed priming resulted in root length decline of ~ 9, ~ 21, ~ 27% in Punjab Canola while ~ 4, ~ 11 and ~ 16% than NP respectively in Faisal Canola (Fig. 1D).

### Shoot and Root fresh or dry weight

**Figure 2:**
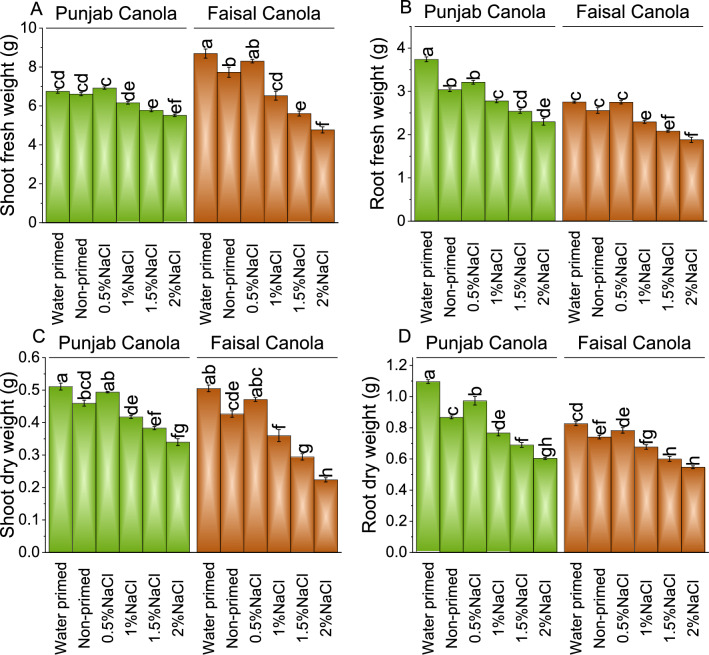
Effect of different pre-treatments of NaCl on shoot fresh weight (**A**), root fresh weight (**B**), shoot dry weight (**C**), and root dry weight (**D**) of Punjab Canola and Faisal Canola. Bars are means of 6 replicates ± SE. Difference letters on bars showed significant changes at *p* ≤ 0.05; Tukey test.

In Punjab Canola, at 0.5% NaCl, plants displayed a ~ 3% increase over WP but a ~ 5% increase over NP plants in shoot fresh weight. As NaCl concentrations increased to 1.0, 1.5, and 2.0%, shoot length was decreased to ~ 9, ~ 14, and ~ 18% compared to WP, and ~ 7, ~ 13, and ~ 16% compared to NP, respectively in Punjab Canola. In Faisal Canola under 0.5% NaCl seed priming a ~ 4% decrease over WP and a ~ 7% increase over NP was noted. However, shoot fresh weight declined with 1.0, 1.5, and 2.0% NaCl seed priming, i.e., ~ 25, ~ 35, and ~ 45% over WP, and ~ 16, ~ 27, and ~ 38% than NP, respectively (Fig. 2A).

Punjab Canola plants under 0.5% NaCl seed priming showed a ~ 14% decrease but a ~ 6% increase in root fresh weight over WP and NP, respectively, in root fresh weight. At 1.0, 1.5, and 2.0% NaCl seed priming, root fresh weight was decreased in Punjab Canola plants to ~ 26, ~ 32, and ~ 39% compared to WP, and ~ 9, ~ 16, and ~ 24% compared to NP, respectively. Under 0.5% NaCl stress, Faisal Canola plants resulted in no change over WP while a ~ 7% increase over NP in root fresh weight. Treatments 1.0, 1.5, and 2.0% NaCl seed priming resulted in the decrease of root fresh weight ~ 17, ~ 24, and ~ 32% over WP and ~ 10, ~ 19, and ~ 26% compared to NP, respectively (Fig. 2B).

Punjab Canola treated with 0.5% NaCl, Punjab Canola plants showed a ~ 3% decrease compared to WP and a ~ 7% increase over NP plants in shoot dry weight. Under 1.0, 1.5, and 2.0% NaCl seed treatment, shoot dry weights were decreased to ~ 18, ~ 25, and ~ 34% over WP, and ~ 9, ~ 17, and ~ 26% than NP, respectively in Punjab Canola. At 0.5% NaCl stress, Faisal Canola showed a ~ 7% decrease from WP and a ~ 10% increase over NP plants. In the case of 1.0, 1.5, and 2.0% NaCl seed priming, a decrease of ~ 29, ~ 42, and ~ 56% compared to WP, and ~ 16, ~ 31, and ~ 48% over NP was noted in Faisal Canola shoot dry weight, respectively (Fig. 2C).

Results showed that at 0.5% NaCl, Punjab Canola plants showed a ~ 11% decrease over WP and ~ 12% increase over NP for root dry weight. As NaCl concentrations increased i.e., 1.0, 1.5, and 2.0% for seed priming, root dry weights decreased to 0.77, 0.69, and 0.60 g, respectively, indicating percentage decreases of ~ 30, ~ 37, and ~ 45% compared to WP, and ~ 12, ~ 20, and ~ 30% over NP, respectively. Similarly, in Faisal Canola 0.5% NaCl seed priming caused ~ 5% decrease from WP but a ~ 6% increase than NP. However, root dry weight was decreased with increasing NaCl concentrations i.e., 2.0% NaCl, decreases of ~ 34% than WP and ~ 26% compared to NP was noted (Fig. 2D).

### Silique length, Silique per plant, seeds per silique, and Branches per plant

**Figure 3:**
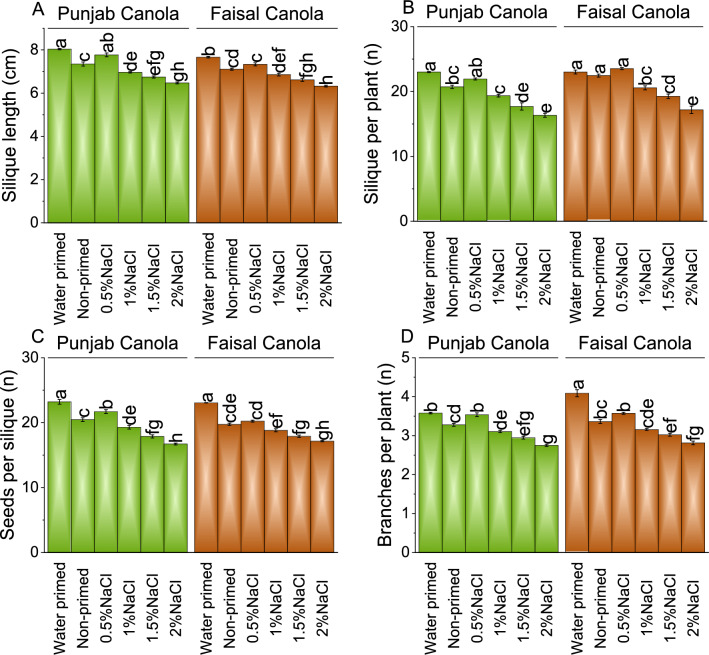
Effect of different pre-treatments of NaCl on silique length (**A**), silique per plant (**B**), seeds per silique (**C**), and branches per plant (**D**) of Punjab Canola and Faisal Canola. Bars are means of 6 replicates ± SE. Difference letters on bars showed significant changes at *p* ≤ 0.05; Tukey test.

For Punjab Canola, when subjected to 0.5% NaCl, silique length showed ~ 3% decrease compared to the WP, while compared to NP, exhibit ~ 6% rise. With increasing salt concentrations, the silique length showed 13, 16, and 19% decrease by adding 1.0, 1.5, and 2.0% NaCl respectively, compared to WP, and ~ 5, ~ 8, and ~ 12% decrease over NP. Similarly, for Faisal Canola, exposure to 0.5% NaCl resulted in a ~ 4% decrease than WP and a 3% increase compared to NP. Adding 1.0, 1.5, and 2.0% NaCl, the silique length showed ~ 11, ~ 14, and ~ 17% decrease over WP, and ~ 4, ~ 7, and ~ 11% decreases compared to NP in Faisal Canola (Fig. 3A).

In Punjab Canola adding 1.0, 1.5, and 2.0% NaCl, there was a ~ 5, ~ 16, ~ 23, and ~ 29% decrease in silique per plant compared to the WP and ~ 6% increase, ~ 7, ~ 15, and ~ 21% decrease over the NP. For Faisal Canola, compared to the WP, there was an increase in silique per plant by ~ 2% under 0.5% NaCl treatment but decreases by ~ 11, ~ 16, and ~ 25% under 1.0, 1.5, and 2.0% NaCl treatments respectively. Compared to the NP, there was an increase in silique per plant by ~ 5% under 0.5% NaCl treatment but decreases by ~ 8, ~ 14, and ~ 24% under 1.0, 1.5, and 2.0% NaCl treatments respectively in Faisal Canola (Fig. 3B).

In Punjab Canola, when subjected to 0.5% NaCl, there was a decrease in plant seed per silique of 6% compared to WP and an increase of 6% compared to the NP. With 1.0, 1.5, and 2.0% NaCl, there was a significant ~ 17, ~ 23, and ~ 28% decrease in plant seed per silique compared to WP and ~ 16, ~ 13, and ~ 18% than NP in Punjab Canola. Under 0.5% NaCl, seed per silique showed a ~ 12% decrease compared to WP and a slight increase of 2% compared to NP in Faisal Canola. With 1.0% NaCl, seed per silique showed ~ 18% decrease compared to WP and ~ 5% compared to NP in Faisal Canola. As salinity levels rose to 1.5% and 2.0% NaCl, the seed per silique showed ~ 22 and ~ 26% decrease compared to WP and ~ 9 and ~ 13% compared to NP, respectively, in Faisal Canola (Fig. 3C).

For Punjab Canola, the branches per plant showed a ~ 1% decrease compared to the WP and a ~ 8% increase over NP. Salinity levels increased to 1.0, 1.5, and 2.0% NaCl, there were significant ~ 13, ~ 18, and ~ 23% decreases in branches per plant respectively than the WP, and by ~ 5, ~ 10, and ~ 16%, respectively, over NP in Punjab Canola.

In Faisal Canola, under WP, 0.5% NaCl, showed ~ 13% decrease in branches per plant from WP but a 6% increase over NP. Subsequent increases in salinity levels to 1.0, 1.5, and 2.0% NaCl led to further decreases in branches per plant, with percentages dropping by ~ 23, ~ 26, and ~ 31%, respectively, compared WP, and by ~ 6, ~ 10, and ~ 16%, respectively, over NP in Faisal Canola (Fig. 3D).

### Chlorophyll content (a, b, and total) and carotenoid

**Figure 4:**
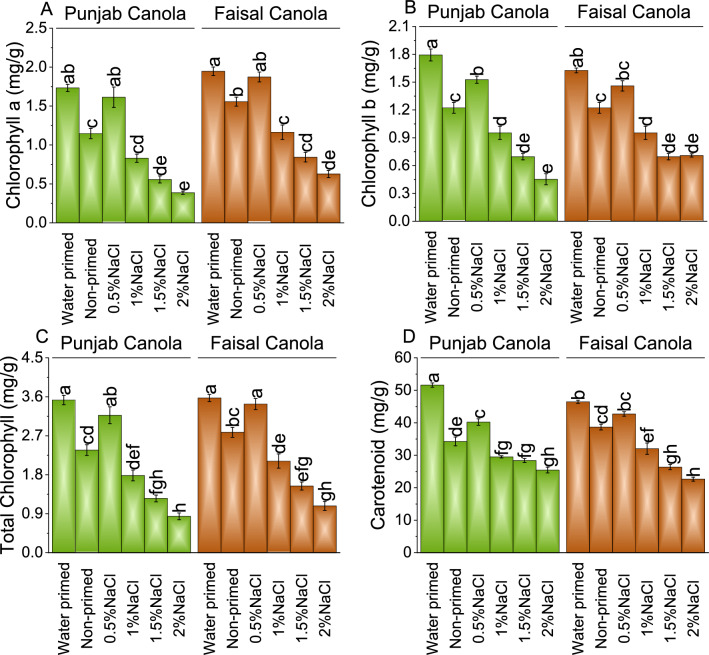
Effect of different pre-treatments of NaCl on chlorophyll a (**A**), chlorophyll b (**B**), total chlorophyll (**C**), and carotenoids (**D**) of Punjab Canola and Faisal Canola. Bars are means of 6 replicates ± SE. Difference letters on bars showed significant changes at *p* ≤ 0.05; Tukey test.

In Punjab Canola, exposure to 0.5% NaCl induced a moderate decrease of ~ 7% in chlorophyll a compared to WP, while exhibiting a notable ~ 41% increase compared to NP. However, as salt concentrations increased, the chlorophyll a content experienced significant declines, with 1.0, 1.5, and 2.0% NaCl treatments resulting in substantial decreases of ~ 52, ~ 68, and ~ 78% from WP, and ~ 28, ~ 51, and ~ 66% observed over NP. Under 0.5% NaCl treatment, chlorophyll a content decreased by ~ 4% compared to WP yet demonstrated a ~ 20% increase relative to NP. Chlorophyll a content decreases by ~ 40, ~ 57, and ~ 68% under 1.0, 1.5, and 2.0% NaCl treatments respectively, compared to WP, and 25, 46, and 60% over the NP (Fig. 4A).

In the presence of 0.5% NaCl, there was a ~ 15% decrease in chlorophyll b compared to WP, while showing a notable ~ 25% increase compared to NP. However, adding 1.0, 1.5, and 2.0% NaCl, the chlorophyll b content decreased by ~ 47, ~ 61, and ~ 75%, respectively, compared to WP, and 22, 43, and 63% decrease than NP. In Faisal Canola, at 0.5% NaCl, there was a significant decrease of ~ 10% compared to WP, and a modest increase of ~ 19% over NP. With 1.0, 1.5, and 2.0% NaCl, there were substantial decrease of ~ 41, ~ 57, and ~ 56%, compared to WP, and 22, 43% and 42% decrease than NP (Fig. 4B).

In Punjab Canola, under the influence of 0.5% NaCl, there is a ~ 10% decrease in total chlorophyll over WP, and a notable 34% increase from NP. As the salinity increases to 1.0, 1.5, and 2.0% NaCl, there are significant ~ 49, ~ 64, and ~ 76% decreases in chlorophyll content than WP and ~ 25, ~ 47, and ~ 65% decrease over the NP. Similarly, in Faisal Canola, the introduction of 0.5% NaCl leads to a ~ 4% decrease compared to WP but a ~ 24% increase compared to NP. However, as salinity concentration increases 1.0, 1.5, and 2.0% NaCl, resulting ~ 41, ~ 57, and ~ 70% decrease in total chlorophyll content and ~ 25, ~ 45, and ~ 61% decrease compared to NP (Fig. 4C).

For Punjab Canola, when subjected to 0.5% NaCl treatment, there was a ~ 22% decrease in carotenoid content compared to WP, but a ~ 17% increase compared to NP. Adding 1.0, 1.5, and 2.0% NaCl, there was a ~ 43, ~ 45, and ~ 51% decrease in carotenoid than WP and ~ 14, ~ 17, and ~ 26% decrease over NP. Moving to Faisal Canola, applying 1.0, 1.5, and 2.0% NaCl, carotenoid content showed ~ 31, ~ 43, and ~ 51% decrease from WP, and ~ 17, ~ 32, and ~ 41% decrease than NP (Fig. 4D).

### Seed yield per plant, moisture content, LRWC, Oil content, and linolenic acid

For Punjab Canola, adding 0.5% NaCl led to a ~ 1% decrease in seed yield per plant compared to the WP group, while compared to the NP group, it showed an ~ 7% rise. Applying 1.0, 1.5, and 2.0% NaCl, there was a significant ~ 14, ~ 23, and ~ 31% decrease in seed yield per plant over WP and ~ 7, ~ 17, and ~ 25% decrease from NP in Punjab Canola. In Faisal Canola, applying 0.5% NaCl, there was a slight decrease of ~ 9% compared to WP, but a significant increase of ~ 3% compared to NP. However, as the NaCl concentration increased by 1.0, 1.5, and 2.0% NaCl, there was significant ~ 15, ~ 18, and ~ 20% decreases in seed yield per plant respectively compared to WP, ~ 4, ~ 7 and ~ 10% decrease compared to NP in Faisal Canola (Table 1).

**Table 1:** Effect of different pre-treatments of NaCl water priming on Seed yield per plant, Moisture content, LRWC, Oil content, oleic acid, and Linolenic acid) of Punjab Canola and Faisal Canola.

Treatments	Punjab Canola	Faisal Canola	Punjab Canola	Faisal Canola	Punjab Canola	Faisal Canola
	Seed yield per plant (n)		Moisture content (%)		LRWC (%)	
Water primed	4.10 ± 0.03a	3.043 ± 0.06de	4.91 ± 0.06a	4.89 ± 0.01a	81.60 ± 0.67a	78.49 ± 0.60c
Non-primed	3.77 ± 0.04 b	2.70 ± 0.01de	4.69 ± 0.02 bcd	4.82 ± 0.03ab	79.69 ± 0.04 b	76.19 ± 0.18 d
0.5%NaCl	4.04 ± 0.07 a	2.77 ± 0.01d	4.86 ± 0.06 a	4.91 ± 0.01 a	81.88 ± 0.04 a	79.17 ± 0.09 b
1%NaCl	3.52 ± 0.10 b	2.60 ± 0.02def	4.54 ± 0.04 de	4.69 ± 0.02 bc	79.49 ± 0.04 b	74.92 ± 0.71 e
1.5%NaCl	3.14 ± 0.06c	2.51 ± 0.00ef	4.36 ± 0.03f	4.60 ± 0.02 cde	79.30 ± 0.04 b	72.73 ± 0.33f
2%NaCl	2.84 ± 0.08d	2.42 ± 0.01f	4.26 ± 0.02f	4.51 ± 0.02 e	77.10 ± 0.04 c	70.46 ± 0.29g

Treatments	Punjab Canola	Faisal Canola	Punjab Canola	Faisal Canola	Punjab Canola	Faisal Canola
	Oil content (%)		Oleic acid (%)		Linolenic acid (%)	
Water primed	42.42 ± 0.11a	40.53 ± 0.26e	60.65 ± 0.60a	53.68 ± 0.48d	12.00 ± 0.16a	11.13 ± 0.06bc
Non-primed	41.90 ± 0.12a	41.21 ± 0.36bcd	58.95 ± 0.14b	52.07 ± 0.17fg	11.36 ± 0.16b	10.79 ± 0.04cd
0.5%NaCl	42.32 ± 0.09ab	42.37 ± 0.28a	60.16 ± 0.08a	52.99 ± 0.17ef	11.93 ± 0.10a	10.98 ± 0.02bc
1%NaCl	41.25 ± 0.09bc	40.25 ± 0.16de	56.87 ± 0.24c	50.99 ± 0.16gh	10.68 ± 0.16cde	10.55 ± 0.04cde
1.5%NaCl	40.73 ± 0.11cde	39.26 ± 0.25fg	55.39 ± 0.33d	49.91 ± 0.28 h	10.08 ± 0.12 fg	10.38 ± 0.04def
2%NaCl	40.20 ± 0.08ef	38.51 ± 0.18f	53.30 ± 0.33e	48.42 ± 0.30i	9.61 ± 0.09g	10.25 ± 0.02ef

Values are the mean of 6 replicates ± SE. Different letters showed significant changes at *p* ≤ 0.05; Tukey Test. Leaf relative water content (LRWC).

At 0.5% NaCl, the plant height showed ~ 1% decrease in moisture content compared to WP plants, but a ~ 4% increase compared to NP in Punjab Canola. However, at 1.0, 1.5, and 2.0% NaCl seed priming, the moisture content declined up to ~ 8, ~ 11, and ~ 13% compared to WP, and ~ 3, ~ 7, and ~ 9% compared to NP, respectively in Punjab Canola. In contrast, Faisal Canola, 0.5% NaCl, WP showed decrease of 0.41% compared to WP, but a ~ 2% increase over NP. Under 1.0, 1.5, and 2.0% NaCl, the moisture content was declined ~ 4, ~ 6, and ~ 8% than WP, and ~ 3, ~ 5, and ~ 6% compared to NP, respectively (Table 1).

Punjab Canola treated with 0.5% NaCl, WP-treated Punjab Canola plants showed a 0.34% increase compared to WP and ~ 3% increase over NP plants in LRWC. Under 1.0, 1.5, and 2.0% NaCl seed treatment, LRWC was decreased to ~ 3, ~ 3, and ~ 6% over WP, and 0.25, 0.49, and ~ 3% than NP, respectively in Punjab Canola. At 0.5% NaCl stress, Faisal Canola showed ~ 1% increase in LRWC from WP while ~ 4% increase over NP plants. In case of 1.0%, 1.5%, and 2.0% NaCl seed priming, a decreases of ~ 5, ~ 7, and ~ 10% compared to WP, and ~ 2, ~ 5, and ~ 8% over NP was noted in Faisal Canola LRWC, respectively (Table 1).

In Punjab Canola, when subjected to 0.5% NaCl, there was a 0.2% decrease in oil content compared to WP and an increase of ~ 1% compared to the NP. With 1.0%, 1.5%, and 2.0% NaCl, there was a significant ~ 3, ~ 4, and ~ 5% decrease in oil content compared to WP and ~ 2, ~ 3, and ~ 4% than NP in Punjab Canola. Under 0.5% NaCl, oil content showed ~ 5% increase compared to WP and a slight increase of ~ 3% compared to NP in Faisal Canola. With 1.0% NaCl, seed per silique showed ~ 1% decrease compared to WP and ~ 2% compared to NP in Faisal Canola. As salinity levels rose to 1.5% and 2.0% NaCl, the oil content showed ~ 3 and ~ 5% decrease compared to WP and ~ 5 and ~ 7% compared to NP, respectively in Faisal Canola (Table 1).

Punjab Canola treated with 0.5% NaCl, WP-treated Punjab Canola plants showed a 0.8% decrease compared to WP and ~ 2% increase over NP plants in oleic acid. Under 1.0%, 1.5%, and 2.0% NaCl seed treatment, oleic acid was decreased to ~ 6, ~ 9, and ~ 12% over WP, and ~ 4, ~ 6, and ~ 10% than NP, respectively in Punjab Canola. At 0.5% NaCl stress, Faisal Canola showed ~ 1% decrease in oleic acid from WP while ~ 2% increase over NP plants. In case of 1.0, 1.5, and 2.0% NaCl seed priming, the oleic acid decreases of ~ 5, ~ 7, and ~ 10% compared to WP, and ~ 2, ~ 4, and ~ 7% over NP was noted in Faisal Canola oleic acid, respectively (Table 1).

For Punjab Canola, when subjected to 0.5% NaCl treatment, there was a 0.6% decrease in linolenic acid compared to WP, but a ~ 5% increase compared to NP. Adding 1.0, 1.5, and 2.0% NaCl, there was a ~ 11, ~ 16, and ~ 20% decrease in linolenic acid than WP and ~ 6, ~ 11, and ~ 15% decrease over NP in Punjab Canola. Moving to Faisal Canola, applying 1.0, 1.5, and 2.0% NaCl, linolenic acid showed ~ 5, ~ 7, and ~ 8% decrease from WP, and ~ 2, ~ 4, and ~ 5% decrease than NP (Table 1).

### Convex hull and hierarchical cluster analysis

**Figure 5:**
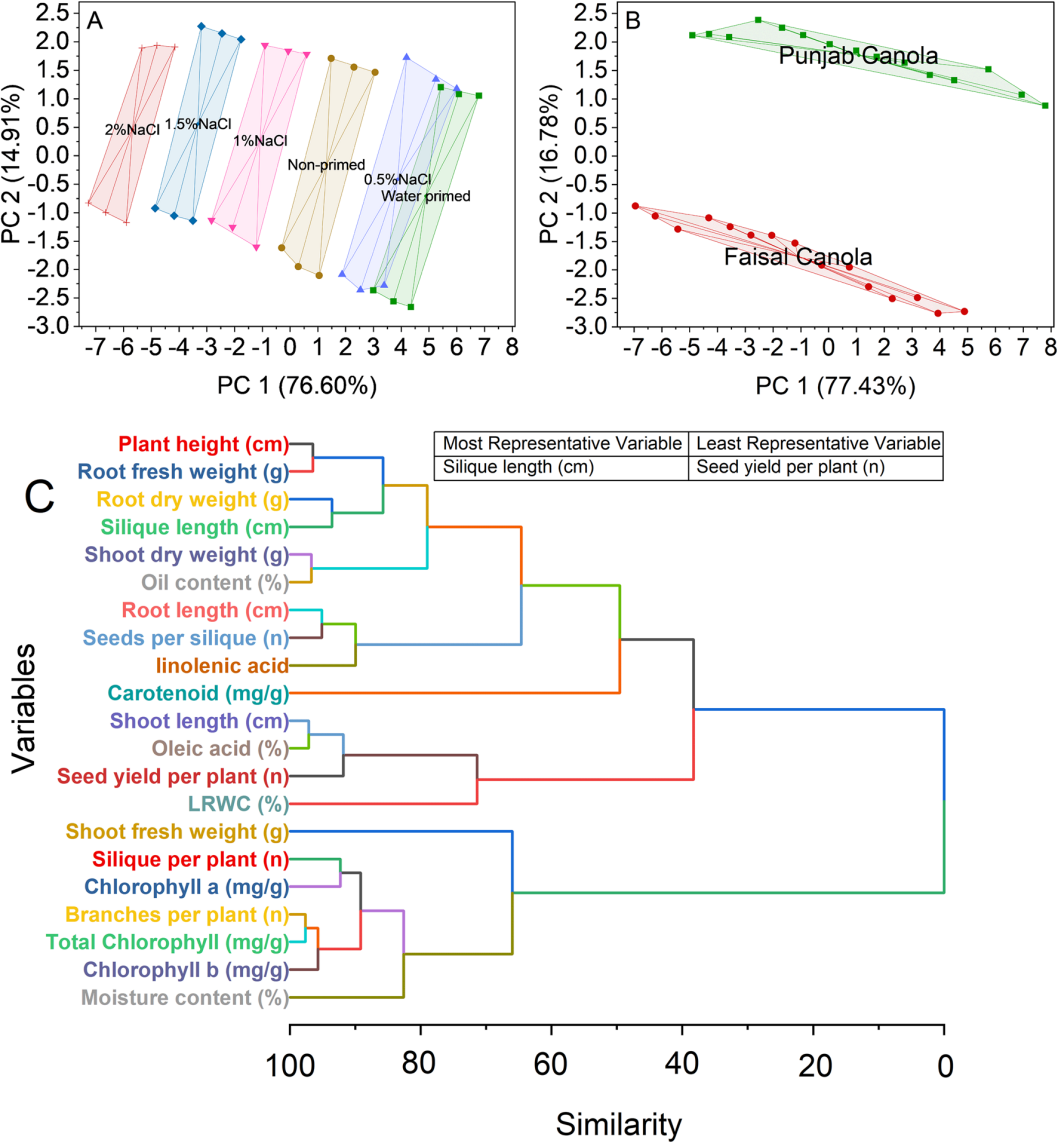
Cluster plot convex hull for treatments (**A**), variety (**B**), and hierarchical cluster plot (**C**) for studied attributes.

The results revealed five distinct treatment groups: non-primed, 0.5%NaCl, 1%NaCl, 1.5%NaCl, and 2%NaCl, each characterized by a unique convex hull encompassing data points. For instance, the convex hull for treatment non-primed included points such as (4.52356, 1.32682) and (2.28901, − 2.50472), while the convex hull for Treatment 0.5%NaCl encompassed points like (7.80192, 0.88075) and (3.19381, − 2.48701). Similarly, convex hulls were identified for Treatments 1%NaCl, 1.5%NaCl, and 2%NaCl (Fig. 5A).

Punjab Canola dominates the analysis, covering an extensive 95.12% of the total area within the convex hull. This indicates that the data points belonging to Punjab Canola are closely clustered and form a cohesive group. On the other hand, Variety Faisal Canola occupies a much smaller portion, accounting for only 4.88% of the total area. (Fig. 5B).

The hierarchical cluster analysis results reveal that variables 18 and 19 have a similarity of 2.40209, suggesting a strong similarity between them. Similarly, variables 11 and 12 share a similarity of 2.89073. Additionally, the table concludes with variable 41, indicating the end of the data (Fig. 5C).

### Pearson correlation analysis

**Figure 6:**
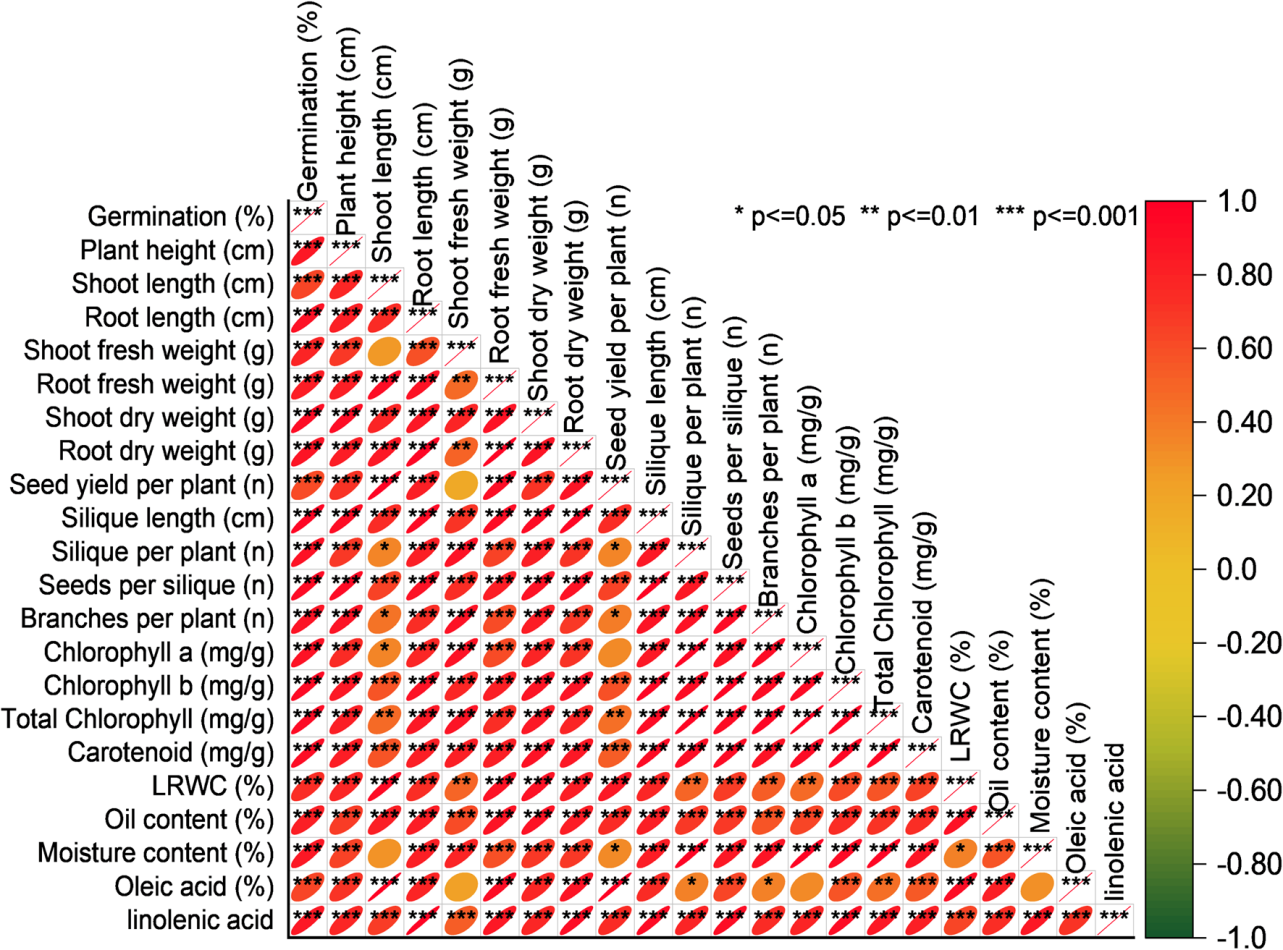
Pearson correlation for studied attributes.

The Pearson correlation analysis reveals that plant height (cm) displays strong positive correlations with both shoot length (cm) and root length (cm), indicating that as the plant’s height increases, so do the shoot and root lengths. Additionally, the positive correlation between shoot fresh weight (g) and root fresh weight (g) suggests that an increase in shoot fresh weight is associated with a corresponding increase in root fresh weight. Seed yield per plant (n) exhibits strong positive correlations with shoot length (cm) and silique length (cm), implying that higher seed yields are linked to longer shoots and siliques. Moreover, the analysis reveals strong positive correlations within the chlorophyll-related variables, including chlorophyll a (mg/g), chlorophyll b (mg/g), and total chlorophyll (mg/g), suggesting that higher levels of total chlorophyll are associated with higher levels of both chlorophyll a and b. Additionally, there is a significant positive correlation between oleic acid (%) and linolenic acid, indicating that an increase in oleic acid is correlated with a rise in linolenic acid content (Fig. 6).

## Discussion

### Low concentration NaCl priming

NaCl can act as a priming agent at low concentrations, effectively breaking seed dormancy and promoting water uptake by seeds^23^. This increased hydration facilitates the activation of enzymatic processes involved in germination, such as the breakdown of stored reserves and the initiation of cell division and elongation^24^. Additionally, low levels of NaCl can induce mild stress responses in seeds, triggering the expression of stress-related genes and the synthesis of protective compounds, which may enhance seed vigour and resilience to adverse environmental conditions^25^. Under moderate stress conditions, such as those induced by low concentrations of NaCl, plants often increase chlorophyll synthesis as a compensatory mechanism to optimize photosynthetic efficiency. Enhanced chlorophyll content facilitates greater light absorption and utilization, promoting carbon assimilation and biomass accumulation, ultimately contributing to improved seed yield per plant^26^. Additionally, salinity caused a significant improvement in oleic acid (C18:1), linolenic acid, stearic acid, palmitic acid, and α- and γ-tocopherols^27,28^.

### High concentration NaCl priming

Conversely, NaCl exerts a more pronounced osmotic stress at higher concentrations on seeds, resulting in reduced water availability and impaired hydration^29^. This osmotic stress can disrupt cellular processes essential for germination, such as enzyme activity, protein synthesis, and membrane integrity^30^. Furthermore, excessive sodium uptake at high NaCl concentrations can lead to ion toxicity, causing imbalances in cellular ion concentrations and metabolic disturbances^31^. These physiological disruptions ultimately hinder seed germination and seedling growth, decreasing vigor and vitality. Furthermore, high salinity disrupts cellular homeostasis and metabolic processes, leading to chlorophyll degradation and impaired photosynthetic activity^32,33^. Osmotic stress and ion toxicity can impede the transport of assimilates and precursors necessary for oil biosynthesis, leading to decreased oil contents in seeds^34^.

### Hydropriming as a better priming agent

Regarding chlorophyll content, hydro priming promotes optimal hydration of seeds, facilitating the activation of metabolic pathways involved in chlorophyll biosynthesis^35^. Hydro priming typically results in enhanced chlorophyll accumulation due to the absence of stress-induced metabolic alterations. This improves photosynthetic efficiency and greater light capture, ultimately contributing to higher chlorophyll contents and enhanced plant vigour^36^. Similarly, hydropriming promotes the synthesis and accumulation of oil in seeds through favorable hydration conditions without the imposition of osmotic stress^37^. Hydro priming facilitates the optimal functioning of lipid biosynthesis pathways by providing adequate moisture for seed imbibition and metabolic activities, leading to increased oil content in seeds^38^. Moreover, hydro priming is advantageous for seed yield per plant as it promotes uniform germination and seedling establishment without the negative impacts of salt stress. By ensuring consistent and rapid seed germination, hydro priming minimizes the likelihood of seedling mortality and enhances stand establishment, thereby maximizing the potential for higher seed yield per plant^39^.

## Conclusion

In conclusion, hydro priming is better than NaCl priming for improving the germination, growth, oil and yield attributes of canola plants. In different levels of NaCl priming, using 0.5%NaCl concentration as priming amendment has potential to improve the canola growth and yield attributes compared to 1.0, 1.5 and 2.0% NaCl priming levels. Growers are recommended to use either 0.5%NaCl or hydropriming for achievement of maximum benefits from priming technology when cultivated canola crop. More investigations at field level, is suggested for declaration of 0.5%NaCl and hydropriming as best amendment for optimization of growth and yield in different oil seed crops.

## Data Availability

All data generated or analyzed during this study are included in this published article.
